# *N*′-(2-Methoxy­benzyl­idene)nicotinohydrazide

**DOI:** 10.1107/S1600536810003831

**Published:** 2010-02-06

**Authors:** Ping Wang, Cong Li, Yong-Qing Su

**Affiliations:** a Faculty of Chemistry and Chemical Engineering, Yunnan Normal University, Kunming 650092, People’s Republic of China

## Abstract

The title compound, C_14_H_13_N_3_O_2_, was prepared by the reaction of 2-methoxy­benzyaldehyde with nicotinic acid hydrazide in methanol. The dihedral angle between the benzene and pyridine rings is 5.9 (3)°. In the crystal structure, mol­ecules are linked by inter­molecular N—H⋯O hydrogen bonds, leading to the formation of chains along the *c* axis; adjacent chains are linked *via* C—H⋯O and C—H⋯N hydrogen bonds.

## Related literature

For general background to Schiff base compounds, see: Archibald *et al.* (1994 ▶); Harada *et al.* (1999 ▶); Ogawa *et al.* (1998 ▶). For related structures, see: Mohd Lair *et al.* (2009 ▶); Sun *et al.* (2009 ▶); Wen *et al.* (2009 ▶).
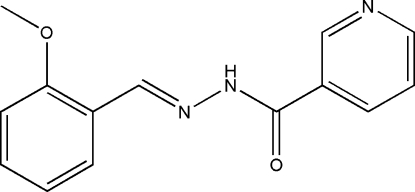


## Experimental

### 

#### Crystal data

C_14_H_13_N_3_O_2_
*M* *_r_* = 255.27
Tetragonal, 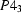

*a* = 9.3264 (13) Å
*c* = 15.594 (3) Å
*V* = 1356.4 (4) Å^3^
*Z* = 4
Mo *K*α radiation
μ = 0.09 mm^−1^
*T* = 298 K
0.20 × 0.20 × 0.18 mm

#### Data collection

Bruker APEXII CCD area-detector diffractometer
Absorption correction: multi-scan (*SADABS*; Sheldrick, 2004 ▶) *T*_min_ = 0.983, *T*_max_ = 0.985
6623 measured reflections
1519 independent reflections
1313 reflections with *I* > 2σ(*I*)
*R*_int_ = 0.028

#### Refinement

*R*[*F* ^2^ > 2σ(*F* ^2^)] = 0.035
*wR*(*F* ^2^) = 0.084
*S* = 1.06
1519 reflections
176 parameters
2 restraints
H atoms treated by a mixture of independent and constrained refinement
Δρ_max_ = 0.09 e Å^−3^
Δρ_min_ = −0.14 e Å^−3^

### 

Data collection: *APEX2* (Bruker, 2004 ▶); cell refinement: *SAINT* (Bruker, 2004 ▶); data reduction: *SAINT*; program(s) used to solve structure: *SHELXS97* (Sheldrick, 2008 ▶); program(s) used to refine structure: *SHELXL97* (Sheldrick, 2008 ▶); molecular graphics: *SHELXTL* (Sheldrick, 2008 ▶); software used to prepare material for publication: *SHELXTL*.

## Supplementary Material

Crystal structure: contains datablocks global, I. DOI: 10.1107/S1600536810003831/ci5028sup1.cif

Structure factors: contains datablocks I. DOI: 10.1107/S1600536810003831/ci5028Isup2.hkl

Additional supplementary materials:  crystallographic information; 3D view; checkCIF report

## Figures and Tables

**Table 1:** Hydrogen-bond geometry (Å, °)

*D*—H⋯*A*	*D*—H	H⋯*A*	*D*⋯*A*	*D*—H⋯*A*
N2—H2⋯O2^i^	0.90 (1)	2.05 (2)	2.897 (2)	157 (3)
C4—H4⋯O1^ii^	0.93	2.58	3.469 (3)	160
C11—H11⋯N3^iii^	0.93	2.53	3.429 (4)	164
C13—H13⋯N1^i^	0.93	2.56	3.487 (3)	176

Symmetry codes: (i) 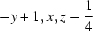; (ii) 
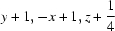
; (iii) 
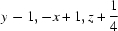
.

**Fig. 1.**
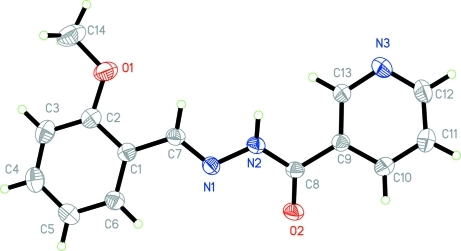
The molecular structure of the title compound, showing the atom-numbering scheme. Displacement ellipsoids are drawn at the 30% probability level.

**Fig. 2.**
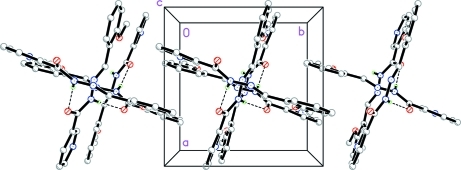
The crystal packing of the title compound. Intermolecular hydrogen bonds are shown as dashed lines.

## supplementary crystallographic information

### Comment

Schiff bases have been received much attention in recent years (Ogawa *et al.*, 1998; Archibald *et al.*, 1994; Harada *et al.*, 1999). As a further investigation of the structures of Schiff base compounds, the title new compound is reported here.

In the title compound, the dihedral angle between the benzene ring and the pyridine ring is 5.9 (3)°. All the bond lengths are comparable with the similar Schiff bases reported previously (Wen *et al.*, 2009; Mohd Lair *et al.*, 2009; Sun *et al.*, 2009).

In the crystal structure, molecules form chains running along the *c* axis through intermolecular N—H···O hydrogen bonds (Table 1 and Fig. 2).

### Experimental

2-Methoxybenzaldehyde (1.0 mmol, 136 mg) and nicotinic acid hydrazide (1.0 mmol, 137 mg) were dissolved in methanol (30 ml). The mixture was stirred at room temperature for 1 h to give a colourless solution. After keeping the solution in air for 3 d, colourless block shaped crystals were formed.

### Refinement

Atom H2 was located in a difference Fourier map and refined isotropically, with the N–H distance restrained to 0.90 (1) Å. The other H atoms were placed in idealized positions and constrained to ride on their parent atoms, with C—H distances in the range 0.93-0.96 Å, and with U_iso_(H) = 1.2 or 1.5U_eq_(C). In the absence of significant anomalous dispersion effects, Friedel pairs were merged before the final refinement.

### Crystal data

**Table:**

C_14_H_13_N_3_O_2_	*D*_x_ = 1.250 Mg m^−^^3^
*M**_r_* = 255.27	Mo *K*α radiation, λ = 0.71073 Å
Tetragonal, *P*4_3_	Cell parameters from 2214 reflections
Hall symbol: P 4cw	θ = 2.5–25.3°
*a* = 9.3264 (13) Å	µ = 0.09 mm^−^^1^
*c* = 15.594 (3) Å	*T* = 298 K
*V* = 1356.4 (4) Å^3^	Block, colourless
*Z* = 4	0.20 × 0.20 × 0.18 mm
*F*(000) = 536	

### Data collection

**Table:**

Bruker APEXII CCD area-detector diffractometer	1519 independent reflections
Radiation source: fine-focus sealed tube	1313 reflections with *I* > 2σ(*I*)
graphite	*R*_int_ = 0.028
ω scans	θ_max_ = 27.0°, θ_min_ = 2.2°
Absorption correction: multi-scan (*SADABS*; Sheldrick, 2004)	*h* = −11→10
*T*_min_ = 0.983, *T*_max_ = 0.985	*k* = −4→11
6623 measured reflections	*l* = −19→19

### Refinement

**Table:**

Refinement on *F*^2^	Primary atom site location: structure-invariant direct methods
Least-squares matrix: full	Secondary atom site location: difference Fourier map
*R*[*F*^2^ > 2σ(*F*^2^)] = 0.035	Hydrogen site location: inferred from neighbouring sites
*wR*(*F*^2^) = 0.084	H atoms treated by a mixture of independent and constrained refinement
*S* = 1.06	*w* = 1/[σ^2^(*F*_o_^2^) + (0.0427*P*)^2^ + 0.084*P*] where *P* = (*F*_o_^2^ + 2*F*_c_^2^)/3
1519 reflections	(Δ/σ)_max_ = 0.001
176 parameters	Δρ_max_ = 0.09 e Å^−^^3^
2 restraints	Δρ_min_ = −0.14 e Å^−^^3^

### Special details

**Table:**

Geometry. All esds (except the esd in the dihedral angle between two l.s. planes) are estimated using the full covariance matrix. The cell esds are taken into account individually in the estimation of esds in distances, angles and torsion angles; correlations between esds in cell parameters are only used when they are defined by crystal symmetry. An approximate (isotropic) treatment of cell esds is used for estimating esds involving l.s. planes.
Refinement. Refinement of *F*^2^ against ALL reflections. The weighted *R*-factor wR and goodness of fit *S* are based on *F*^2^, conventional *R*-factors *R* are based on *F*, with *F* set to zero for negative *F*^2^. The threshold expression of *F*^2^ > σ(*F*^2^) is used only for calculating *R*-factors(gt) etc. and is not relevant to the choice of reflections for refinement. *R*-factors based on *F*^2^ are statistically about twice as large as those based on *F*, and *R*- factors based on ALL data will be even larger.

### Fractional atomic coordinates and isotropic or equivalent isotropic displacement parameters (Å^2^)

**Table:**

	*x*	*y*	*z*	*U*_iso_*/*U*_eq_	
N1	0.54565 (17)	0.50653 (18)	0.21981 (11)	0.0443 (4)	
N2	0.41517 (19)	0.53627 (19)	0.18161 (11)	0.0457 (4)	
N3	0.0801 (2)	0.6980 (3)	0.03464 (14)	0.0763 (7)	
O1	0.84079 (19)	0.3686 (2)	0.05708 (12)	0.0681 (5)	
O2	0.33671 (17)	0.66164 (18)	0.29682 (10)	0.0586 (4)	
C1	0.7782 (2)	0.4068 (2)	0.20059 (15)	0.0454 (5)	
C2	0.8837 (2)	0.3667 (2)	0.14119 (15)	0.0508 (5)	
C3	1.0208 (3)	0.3312 (3)	0.1677 (2)	0.0635 (7)	
H3	1.0901	0.3046	0.1279	0.076*	
C4	1.0535 (3)	0.3360 (3)	0.2543 (2)	0.0662 (7)	
H4	1.1455	0.3123	0.2724	0.079*	
C5	0.9519 (3)	0.3753 (3)	0.31421 (19)	0.0621 (6)	
H5	0.9751	0.3784	0.3722	0.075*	
C6	0.8156 (3)	0.4098 (2)	0.28694 (15)	0.0516 (5)	
H6	0.7469	0.4358	0.3273	0.062*	
C7	0.6357 (2)	0.4433 (2)	0.17089 (15)	0.0466 (5)	
H7	0.6091	0.4202	0.1151	0.056*	
C8	0.3205 (2)	0.6206 (2)	0.22245 (13)	0.0420 (5)	
C9	0.1932 (2)	0.6646 (2)	0.17108 (14)	0.0428 (5)	
C10	0.0723 (3)	0.7159 (3)	0.21159 (16)	0.0623 (7)	
H10	0.0690	0.7228	0.2710	0.075*	
C11	−0.0433 (3)	0.7567 (4)	0.1626 (2)	0.0797 (9)	
H11	−0.1265	0.7907	0.1884	0.096*	
C12	−0.0339 (3)	0.7463 (4)	0.07564 (19)	0.0779 (9)	
H12	−0.1125	0.7749	0.0432	0.093*	
C13	0.1914 (3)	0.6590 (3)	0.08281 (14)	0.0571 (6)	
H13	0.2732	0.6259	0.0550	0.069*	
C14	0.9423 (4)	0.3294 (4)	−0.0070 (2)	0.0921 (11)	
H14A	1.0234	0.3926	−0.0041	0.138*	
H14B	0.8987	0.3366	−0.0626	0.138*	
H14C	0.9734	0.2325	0.0026	0.138*	
H2	0.394 (3)	0.496 (3)	0.1309 (11)	0.080*	

### Atomic displacement parameters (Å^2^)

**Table:**

	*U*^11^	*U*^22^	*U*^33^	*U*^12^	*U*^13^	*U*^23^
N1	0.0447 (10)	0.0450 (9)	0.0430 (10)	0.0038 (8)	−0.0060 (8)	−0.0027 (8)
N2	0.0473 (10)	0.0510 (10)	0.0387 (10)	0.0048 (8)	−0.0067 (8)	−0.0073 (8)
N3	0.0612 (14)	0.118 (2)	0.0500 (13)	0.0151 (13)	−0.0096 (11)	0.0096 (13)
O1	0.0652 (10)	0.0875 (12)	0.0516 (10)	−0.0048 (9)	0.0080 (9)	−0.0131 (9)
O2	0.0685 (10)	0.0673 (10)	0.0399 (8)	0.0156 (8)	−0.0110 (8)	−0.0140 (8)
C1	0.0491 (12)	0.0404 (11)	0.0468 (12)	−0.0005 (9)	0.0015 (9)	−0.0005 (9)
C2	0.0522 (12)	0.0462 (11)	0.0538 (14)	−0.0060 (9)	0.0038 (10)	−0.0048 (10)
C3	0.0490 (13)	0.0615 (14)	0.0799 (18)	−0.0002 (11)	0.0098 (13)	−0.0091 (13)
C4	0.0479 (13)	0.0646 (16)	0.086 (2)	0.0025 (11)	−0.0054 (14)	0.0038 (14)
C5	0.0647 (15)	0.0641 (15)	0.0576 (15)	0.0002 (12)	−0.0114 (13)	0.0085 (12)
C6	0.0554 (13)	0.0517 (12)	0.0478 (13)	0.0016 (10)	0.0009 (10)	0.0040 (10)
C7	0.0519 (12)	0.0485 (11)	0.0395 (10)	0.0022 (9)	−0.0030 (10)	−0.0042 (9)
C8	0.0492 (11)	0.0405 (10)	0.0361 (11)	0.0013 (8)	−0.0022 (9)	−0.0018 (8)
C9	0.0449 (11)	0.0421 (10)	0.0414 (11)	−0.0001 (8)	−0.0009 (9)	−0.0020 (9)
C10	0.0606 (16)	0.0807 (17)	0.0458 (14)	0.0162 (12)	0.0040 (12)	−0.0068 (13)
C11	0.0526 (14)	0.111 (2)	0.076 (2)	0.0233 (14)	0.0058 (14)	−0.0014 (19)
C12	0.0502 (15)	0.114 (2)	0.0692 (19)	0.0146 (14)	−0.0098 (13)	0.0141 (17)
C13	0.0467 (13)	0.0809 (16)	0.0439 (13)	0.0107 (11)	0.0008 (10)	0.0058 (11)
C14	0.080 (2)	0.127 (3)	0.069 (2)	−0.0180 (19)	0.0249 (16)	−0.0256 (19)

### Geometric parameters (Å, °)

**Table:**

N1—C7	1.279 (3)	C5—C6	1.379 (3)
N1—N2	1.383 (2)	C5—H5	0.93
N2—C8	1.343 (3)	C6—H6	0.93
N2—H2	0.897 (10)	C7—H7	0.93
N3—C12	1.320 (4)	C8—C9	1.489 (3)
N3—C13	1.332 (3)	C9—C13	1.378 (3)
O1—C2	1.371 (3)	C9—C10	1.378 (3)
O1—C14	1.424 (3)	C10—C11	1.375 (4)
O2—C8	1.231 (2)	C10—H10	0.93
C1—C6	1.391 (3)	C11—C12	1.362 (4)
C1—C2	1.402 (3)	C11—H11	0.93
C1—C7	1.448 (3)	C12—H12	0.93
C2—C3	1.385 (3)	C13—H13	0.93
C3—C4	1.385 (4)	C14—H14A	0.96
C3—H3	0.93	C14—H14B	0.96
C4—C5	1.380 (4)	C14—H14C	0.96
C4—H4	0.93		
			
C7—N1—N2	114.42 (17)	C1—C7—H7	119.3
C8—N2—N1	119.46 (17)	O2—C8—N2	123.23 (19)
C8—N2—H2	120.9 (19)	O2—C8—C9	121.28 (18)
N1—N2—H2	119.6 (19)	N2—C8—C9	115.49 (17)
C12—N3—C13	116.6 (2)	C13—C9—C10	117.4 (2)
C2—O1—C14	118.3 (2)	C13—C9—C8	122.5 (2)
C6—C1—C2	118.0 (2)	C10—C9—C8	120.1 (2)
C6—C1—C7	122.3 (2)	C11—C10—C9	118.9 (2)
C2—C1—C7	119.7 (2)	C11—C10—H10	120.6
O1—C2—C3	123.9 (2)	C9—C10—H10	120.6
O1—C2—C1	115.1 (2)	C12—C11—C10	118.8 (3)
C3—C2—C1	121.0 (2)	C12—C11—H11	120.6
C4—C3—C2	119.1 (2)	C10—C11—H11	120.6
C4—C3—H3	120.4	N3—C12—C11	124.0 (3)
C2—C3—H3	120.4	N3—C12—H12	118.0
C5—C4—C3	121.1 (2)	C11—C12—H12	118.0
C5—C4—H4	119.4	N3—C13—C9	124.3 (2)
C3—C4—H4	119.4	N3—C13—H13	117.9
C6—C5—C4	119.1 (3)	C9—C13—H13	117.9
C6—C5—H5	120.4	O1—C14—H14A	109.5
C4—C5—H5	120.4	O1—C14—H14B	109.5
C5—C6—C1	121.7 (2)	H14A—C14—H14B	109.5
C5—C6—H6	119.2	O1—C14—H14C	109.5
C1—C6—H6	119.2	H14A—C14—H14C	109.5
N1—C7—C1	121.4 (2)	H14B—C14—H14C	109.5
N1—C7—H7	119.3		

### Hydrogen-bond geometry (Å, °)

**Table:**

*D*—H···*A*	*D*—H	H···*A*	*D*···*A*	*D*—H···*A*
N2—H2···O2^i^	0.90 (1)	2.05 (2)	2.897 (2)	157 (3)
C4—H4···O1^ii^	0.93	2.58	3.469 (3)	160
C11—H11···N3^iii^	0.93	2.53	3.429 (4)	164
C13—H13···N1^i^	0.93	2.56	3.487 (3)	176

Symmetry codes: (i) −*y*+1, *x*, *z*−1/4; (ii) *y*+1, −*x*+1, *z*+1/4; (iii) *y*−1, −*x*+1, *z*+1/4.
